# Treatment of Cigarette Butts: Biodegradation of Cellulose Acetate by Rot Fungi and Bacteria

**DOI:** 10.3390/microorganisms12112285

**Published:** 2024-11-11

**Authors:** Rodrigo Morales-Vera, Javiera Cantillana, Félix Arto-Paz, Camila Hernández, Alex Echeverría-Vega, Cristian Valdés

**Affiliations:** 1 Escuela de Ingeniería en Biotecnología, Centro de Biotecnología de los Recursos Naturales (Cenbio), Universidad Católica del Maule, Avda. San Miguel 3605, Talca 3466706, Chile; javiera.herrera.01@alu.ucm.cl (J.C.); chernandezs@ucm.cl (C.H.); 2 Doctorado en Biotecnología Traslacional (DBT), Universidad Católica del Maule, Avda. San Miguel 3605, Talca 3466706, Chile; felix.arto@alumnos.ucm.cl; 3 Centro de Investigación de Estudios Avanzados del Maule, Vicerrectoría de Investigación y Postgrado, Universidad Católica del Maule, Avenida San Miguel 3605, Talca 3466706, Chile; aecheverria@ucm.cl

**Keywords:** cigarette butts, rot fungi, bacteria, biodegradation, biotreatment

## Abstract

This study demonstrated the biodegradation of two different brands of cigarette butts (CBs), which are primarily composed of cellulose acetate, by four distinct microorganisms. These included the white rot fungus *Pleurotus ostreatus*, the brown rot fungus *Lentinus lepideus*, and the bacteria *Bacillus cereus* and *Pseudomonas putida*. After 31 days of treatment, weight loss measurements revealed a mass loss of 24–34%, where *B. cereus* exhibited the greatest efficacy in terms of mass loss for both brands of CBs. Fourier-Transform Infrared Spectroscopy (FTIR), confocal microscopy, and scanning electron microscopy (SEM) confirmed changes in the surface of the CBs, attributable to structural wear and material breakdown, indicating effective biodegradation by the evaluated microorganisms. Furthermore, the analyses confirmed changes in the surface of the CBs, attributable to structural wear and material breakdown, indicating effective biodegradation by the evaluated microorganisms.

## 1. Introduction

Cigarette consumption has become one of the greatest problems worldwide. As described by the World Health Organization (WHO), tobacco represents one of the greatest threats to Earth’s resources because of its effects on the life cycle of the planet’s flora and fauna [1]. Cigarette butts represent one of the most abundant types of waste [2]. In 2020, it was estimated that around 4.78 trillion cigarettes were consumed worldwide. It is projected that in 2025 around 4.55 trillion cigarettes will be consumed globally [3]. It is currently estimated that around 1.2 billion tons of cigarette butts are released into the environment. According to a study carried out by Coastal Clean Up, which was based on data from coastal clean-ups, cigarette butts were among the most frequently thrown waste [4]. Residues from this industry can be found all over the world, generating a global problem since their collection is difficult, and their appropriate final disposal is even more complicated. In urban areas, cigarette butts are heterogeneously distributed. However, it is very common to find them in coastal areas, especially in tourist areas [5]. These can be dispersed by watercourses, lakes, seas, and soil. Their persistence in the environment has toxic effects due to the high presence of various chemical compounds used both in their processing and during their production, including fungicides, herbicides, insecticides, and pesticides, as well as products such as tar, hydrogen cyanide, nitrates, and ammonia, among others [6].

Tobacco additives play an important role in manufacturing and product quality. The main ones are menthol, glycerol, 1,2-propylene glycol, sorbitol, ammonium compounds, and sugars [7]. The high presence of dangerous compounds such as nicotine, heavy metals, polycyclic aromatic hydrocarbons (PAHs), and ethyl phenol, among others, can attack the central nervous system and cause heart conditions due to their easy absorption by various organs [8]. Recent studies have shown that cigarette butts inhibit plant growth. Compounds such as nicotine and PAH have been found in plant products, food crops such as tea, and spices. These products may cause alterations in the chlorophyll content, as well as a decrease in the length of the shoots and roots [9].

Most cigarette filters are composed of up to 12,000 cellulose acetate (CA) fibers [9], which have poor filtration performance for toxic compounds. Once these compounds adhere to the filter, they confer toxicity, affecting biodegradation [10]. In addition, the inefficient final disposal of cigarette butts results in the free dispersion of tobacco residues and a series of compounds, which contaminate watercourses and land resources for a period that is still unknown [9].

Due to its high degree of substitution (DS)—the average number of acetyl groups replacing hydroxyl groups in the anhydroglucose units of cellulose—cellulose acetate can be differentiated from simple cellulose. This, together with the compressed composition of the filters, makes it almost impossible to complete the degradation of cigarette butts under natural conditions [5,11]. Recent studies have shown that the toxicity of smoked cigarettes is greater due to the highly toxic components found [2].

The final disposal of cigarette butts represents a great expense for the world’s governments because of their difficult collection. The fraction that is collected is disposed of in different landfills where it is processed together with the rest of the waste [2]. Therefore, one of the strategies for the potential management of this type of waste is the use of microorganisms, such as fungi and bacteria. Several studies have investigated the ability of these microbes to degrade different polymers through action mechanisms linked to weight loss and mechanical, physical, and chemical properties, among other factors [12].

Wood rot fungi have been used on many occasions to degrade various organic and inorganic compounds [13]. These organisms synthesize intracellular and extracellular enzymes such as amylases, proteases, cellulases, and lipases that can transform any type of substrate into organic substrates, and they participate in the oxidation of some inorganic compounds to assimilate as a carbon source, digesting various polymers [14]. Wood rot fungi are divided into white rot fungi (WRFs) and brown rot fungi (BRFs) [15]. WRFs are distinguished by their exceptional ability to degrade lignocellulosic material, including lignin, cellulose, and hemicellulose, through a variety of enzymes, such as endoglucanases for degrading cellulosic components and lignin peroxidase, manganese peroxidase, and laccase for lignin degradation [16]. BRF also degrades cellulosic components but lacks peroxidases involved in lignin degradation; however, it is decomposed by an oxidative radical system and by carbohydrate-active enzymes (CAZymes) [17]. The enzymatic capacity of both WRF and BRF presents remarkable flexibility in terms of the substrates they can degrade, even being effective in the degradation of synthetic polymers, such as plastics [18,19].

Bacteria, which include *Streptomyces*, *Micrococcus*, *Pseudomonas*, *Rhodococcus*, and *Bacillus*, constitute an important group worth studying regarding the degradation of organic and inorganic polymers. Depending on the species used, several rates of bacterial biodegradation can be observed. *Pseudomonas* is one of the most studied bacteria and one of the most efficient in terms of biodegradation, which may be due to its ability to secrete enzymes such as catalase, protease, gelatinase, cellulase, and pectinase [20]. *Bacillus* is also widely studied for its enzymatic potential to degrade organic compounds and has demonstrated the ability to degrade various petroleum-derived plastics, such as polypropylene (PP) and polyvinyl chloride (PVC), through the release of exocellular enzymes [21].

Biodegradation methods have been rarely studied for treating CA. In 2020, a study evaluated the degradation rates of 14 different cellulose materials during pilot-scale composting. Significant differences in degradation rates were observed depending on the type of cellulose derivative. While cellulose was almost 100% degraded after 4 weeks of composting, CA (DS ~ 2.5) did not degrade during the composting experiments [22]. In another experiment, AC showed no biodegradation measured as the degree of hydrolysis after two days of utilizing an enzyme cocktail consisting of cellulase, mannase, xylanase, and β-glucosidase [23]. The susceptibility of a material to enzymatic or biodegradation can often be enhanced by UV irradiation (as simulated sunlight). A combination of deacetylating enzymes (lipase or esterase) and cellulase did not significantly promote the degradation of CA with DS = 2.4. However, when the same CA was UV irradiated before suspension in sterilized buffer or cellulase solution, 23% and 60% weight loss took place, respectively [24]. Regarding fungi treatments, Pleurotus production using cigarette butts as a substrate found that after a six-week period, fungi species had accelerated biodegradation compared to an estimated natural biodegradation of 25% [25]. It is necessary, then, to study the use of these organisms to carry out substantial degradation of cigarette butts, which could be a feasible strategy for the treatment and final disposal of this pollutant. Consequently, this study analyzed and compared, for first time, four different biological treatments for CA using brown rot fungi (*Lentinus lepideus*), white rot fungi (*Pleurotus ostreatus*) and two well-known bioremediation bacteria, *Bacillus cereus* and *Pseudomonas putida*.

## 2. Materials and Methods

### 2.1. Cigarette Butt Samples

Smoked cigarette filters with no remaining tobacco were obtained from active smokers from Talca City, VII Maule region, Chile. Smokers were provided with cigarettes from two different brands, “Brand 1” and “Brand 2”. The smoked cigarette filters with no remaining tobacco were separated and maintained by brand in hermetic bags.

### 2.2. Inocula Preparation

The fungi inocula of *L. lepideus* and *P. ostreatus* were obtained from the stock samples of the Bioconversion and Bioproducts Laboratory from the Center of Biotechnology of Natural Resources (CenBio) at Universidad Católica del Maule. Approximately 5 × 5 mm was extracted into new Petri dishes with PDA (potato dextrose agar) medium. The bacteria *P. putida* and *B. cereus* provided by the Bioprocesses Laboratory from the CenBio were inoculated into a 4 µL stock sample in 40 mL of CASO broth (soybean casein digest broth). To determine the viability of the bacteria, a growth curve was performed by measuring optical density (OD) at 600 nm in a spectrophotometer obtaining an exponential growth after 4 h. Both the plates and flasks were inoculated in a laminar flow cabinet to avoid contamination. The incubation of fungi was carried out in an incubator at 28 °C. (BJPX-HIV, Biobase, Jinan, Shandong, China) while the bacteria were incubated in a shaker at 32 °C at 200 rpm (JSSI-100C, JSR, Gongju, Republic of Korea).

### 2.3. Preparation of Samples

For both the fungi and bacterial treatment, the cigarette butts were placed in an oven at 80 °C for 48 h; once dried, they were placed in a desiccator for 20 min and then weighed on an analytical balance to obtain their dry weight, obtaining an average of 0.179 g and 0.199 g for Brand 1 and 2, respectively. Next, these samples were sterilized in an autoclave with enough distilled water to obtain 70% moisture content, which was calculated by Equation (1).

(1) $$\%MC=\frac{\left(Ww-Dw\right)}{Ww}\times 100$$

Equation (1). Moisture content of the cigarette samples

Ww: Wet weight of cigarette butts.

Dw: Dry weight of cigarette butts.

% MC: Moisture content percentage.

### 2.4. Biodegradation Test

For the test carried out with rotting fungi, *P. ostreatus* and *L. lepideus* plates fully colonized in PDA media were selected, to which an already conditioned cigarette butt was introduced according to the procedure described above (Section 2.3), this procedure was repeated for each brand of cigarette butt. The filters were introduced into the plates in a laminar flow cabinet and then placed in an incubator at 28 °C for 31 days.

Bacteria experiments for *B. Cereus* and *P. putida* were carried out immediately after the inoculation of 1.37 × 10^2^ and 1.01 × 10^3^ CFU/mL, respectively, introducing the conditioned cigarette butts and later adding them to the shaker model JSSI-100C at 32 °C, 200 rpm for 31 days. In both cases, controls were utilized, consisting of uninoculated smoked cigarette butt filters with culture medium that followed the same treatment. Additional nutrients were not added during the experiments.

### 2.5. Weight Loss

To measure the weight loss of the treated cigarette butts, they were dried in an oven at 80 °C for 48 h; once dried, they were placed in a desiccator for 20 min and then weighed on an analytical balance. The weight obtained was compared with the one initially indicated in the “Preparation of Sample” Section 2.3.

The percentage of weight loss (WL) was determined by the ratio shown in Equation (2).

(2) $$WL\left(\%\right)=\frac{\left(W1-W2\right)}{W1}\times 100$$

Equation (2). Ratio to calculate weight loss percentage for cigarette butts.

W1: Initial weight of cigarette butts.

W2: Final weight of cigarette butts.

WL (%): Percentage of weight loss.

### 2.6. Fourier-Transform Infrared Spectroscopy (FTIR)

For the FTIR analysis, the samples (treated cigarette butts) and controls (cigarette butts without biological treatment) were ground in liquid nitrogen with a previously sterilized mortar. Then, 10 mg of each sample previously dried at 70 °C for 4 h was analyzed using the FTIR technique with Attenuated Total Reflectance (RTA) in a Spectrometer (Nicolet IS 5, Thermo Scientific, Waltham, MA, USA). The measured wavelength range was 450–4000 cm^−1^, where the resolution was 4 cm^−1^, and the number of scans was 32.

### 2.7. Confocal Microscopy

The preparation of the samples treated with rot fungi was carried out through DAPI and SYPRO staining, adhering to the DNA of both active and inactive cells and proteins, respectively. Bacteria-treated samples were treated with propidium iodide and SYTO9, where the first one penetrates broken and damaged cell membranes, while the second one stains all cells. In both cases, a 15 min incubation was carried out in the dark for washing later with 95% alcohol and drying at room temperature. The samples were visualized at 60× in the confocal microscope (Stellaris 5, Leica, Wetzlar, Germany).

### 2.8. Scanning Electron Microscopy (SEM)

The method was based on ASTM E1508 standard “Standard Guide for Quantitative Analysis by Energy-Dispersive Spectroscopy” [26]. For SEM visualization, the morphology of the cellulose acetate particles was observed scanning electron microscope (Zeiss EVO MA10, Oberkochen, Germany).

### 2.9. Statistical Analysis

Analysis of variance was performed on the three replicates in each of the treatments indicated, using the Infostat package v.2020 software, maintaining a 95% certainty level, to compare the effect of rotting fungi and bacteria, in the different brands of cigarette butts. When finding significant differences, a Tukey analysis was performed with the same level of reliability.

## 3. Results

### 3.1. Weight Loss

To quantify the biodegradation of cellulose acetate-based cigarette butts, weight loss was determined as a response variable to biological treatments.

**Figure 1:**
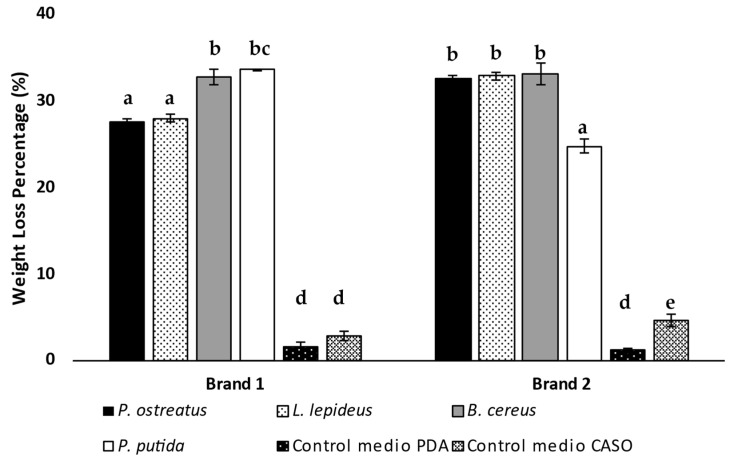
Comparative analysis of the percentage of weight loss in commercial cigarette butts for different treatments conducted with rot fungi and bacteria. The bars indicate the standard error of triplicates and letters that are not equal indicate the presence of significant differences.

Figure 1 shows the percentages of the weight loss of cigarette butts exposed to the action of rot fungi and bacteria. The cigarette butts corresponding to both commercial brands displayed an average biodegradation result treated with bacteria of 31% compared to the average of 30% achieved by rot fungi after 31 days. The control medium (PDA and CASO) showed minimal degradation, confirming that the weight loss is primarily due to microbial activity rather than natural degradation.

When making a comparative analysis of the different treatments for each of the brands of cigarette butts studied, it was observed that for Brand 1, bacterial treatments (*B. cereus* and *P. putida*) resulted in significantly higher biodegradation percentages of 33% and 34%, respectively, compared to the fungi treatments with *P. ostreatus* and *L. lepideus*, which resulted in 27% and 28% respectively. In contrast, for Brand 2, *B. cereus* achieved a higher biodegradation rate of 33%, statistically comparable to the fungi treatments (*P. ostreatus* at 33% and *L. lepideus* at 33%), whereas *P. putida* showed a significantly lower biodegradation rate at 25%. These results demonstrate the biodegradative effect of both the rot fungi and bacteria studied, where the observed differences between them and across the brands evaluated could be related to variations in the structural composition of the cellulose acetate in each brand.

### 3.2. Fourier-Transform Infrared Spectroscopy (FTIR)

**Figure 2:**
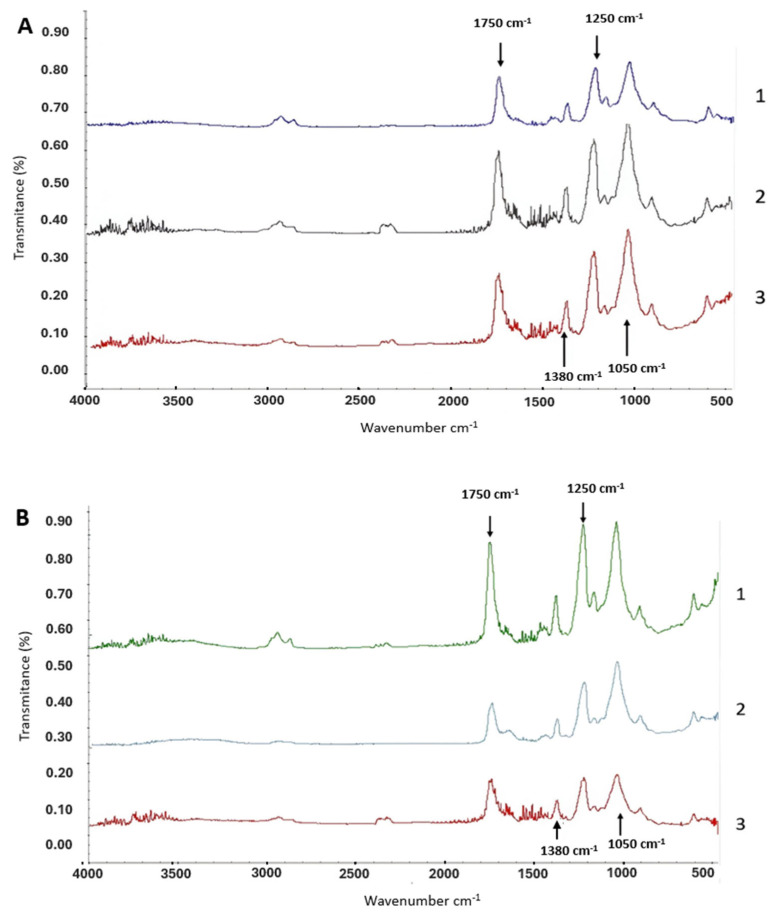

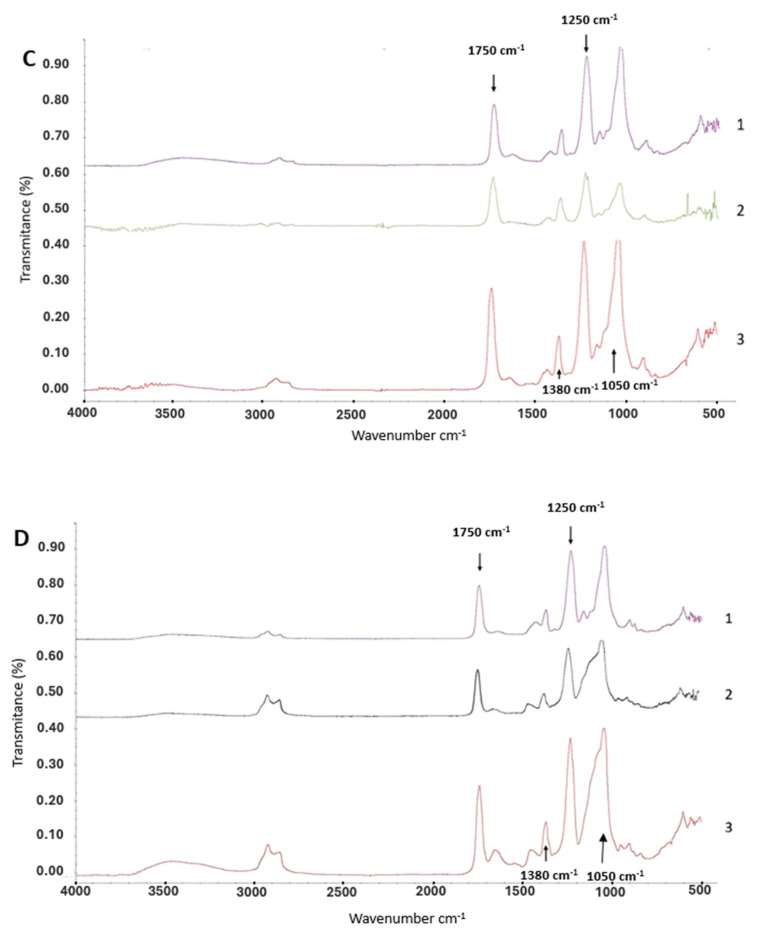
FTIR spectra of cigarette butts treated with rot fungi and bacteria. (**A**) Brand 1 cigarette butts treated with rot fungi (1: control-no biological treatment, 2: treated with *P. ostreatus*, 3: treated with *L. lepideus*). (**B**) Brand 2 cigarette butts treated with rot fungi (1: control-no biological treatment, 2: treated with *P. ostreatus*, 3: treated with *L. lepideus*). (**C**) Brand 1 cigarette butts treated with bacteria (1: control-no biological treatment, 2: treated with *B. cereus*, 3: treated with *P. putida*). (**D**) Brand 2 cigarette butts treated with bacteria (1: control-no biological treatment, 2: treated with *B. cereus*, 3: treated with *P. putida*).

To analyze the chemical modification of the cellulose acetate compound, Fourier-Transform Infrared Spectroscopy (FTIR) analysis was performed for all experiments. Although FTIR is not a quantitative technique, it can give us a trend regarding the change in the functional groups on the surface of the material as a result of the treatments utilized. The analysis revealed distinct changes in the spectra of cigarette butts treated with rot fungi and bacteria, allowing us to observe a substantial degradation of the cellulose acetate compound, particularly in the 2000–700 cm^−1^ zone of the spectrum. In addition, it is possible to observe different peak sizes between brands. Brand 1 showed smaller peaks in the most relevant functional groups within the spectrum 1050 cm^−1^, 1250 cm^−1^, 1380 cm^−1^ and 1750 cm^−1^ (Figure 2).

Shown in Figure 2A, Brand 1 cigarette butts treated with rot fungi exhibited an increase in peak sizes within the spectrum, particularly noticeable at 1750 cm^−1^ (-C=O), which represents the ketone functional group. This increase suggests a higher DS of the cellulose acetate molecule, making the ketone groups more accessible or increased in number, which in turn could make the material more susceptible to breakdown by rot fungi, potentially enhancing its biodegradability. In contrast, Figure 2B shows that Brand 2 butts, subjected to similar fungal treatments, showed an overall reduction in the size of the spectrum. Notable reductions occurred in the 1500–900 cm^−1^ (C-O/carboxylic acid and CH3/alkane functional groups), 1050 cm^−1^ (C-O-C/dialkyl), and 1750 cm^−1^ (-C=O/acetate) regions, indicating a decrease in the degree of acetylation of the cellulose acetate compound, rendering it more prone to degradation, as fewer acetyl groups are available to protect the cellulose chains.

In Figure 2C, Brand 1 cigarette butts treated with *B. cereus* bacteria demonstrated a decrease in peak sizes compared to the control; however, this reduction was not observed in cigarette butts treated with *P. putida*, which instead showed an increase in peak numbers across the 2000–500 cm^−1^ zone (CH_3_/alkane, C-O/carboxylic acid, and C-O-C/dialkyl functional groups). Figure 2D further illustrates that Brand 2 cigarette butts treated with bacteria experienced a decrease in the 2000–500 cm^−1^ zone for samples treated with *B. cereus*, while those treated with *P. putida* showed an increase in this zone. Additionally, increases were noted in the 3000–2700 cm^−1^ zone (carboxylic acids) and in the 3700–3000 cm^−1^ zone (alcohols, aromatic compounds, carboxylic acids), indicating stretching of the nonacetylated cellulose of the molecule.

### 3.3. Confocal Microscopy

To visualize the effects of the treatments used on the cellulose acetate cigarette butts, a series of observations were made through confocal microscopy.

**Figure 3:**
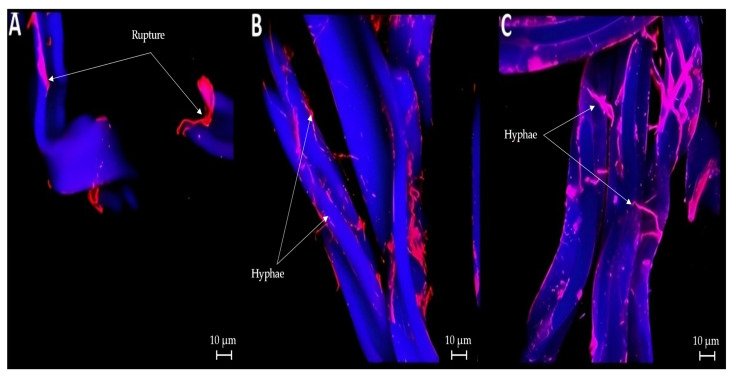
Confocal microscopy of cigarette butts: (**A**) smoked cigarette butt control (no biological treatment); (**B**) smoked cigarette butt treated with *P. ostreatus;* (**C**) smoked cigarette butt treated with *L. lepideus*.

When the samples were stained with DAPI and SYPRO, there was no fungal material in the cellulose acetate strands in the controls (Figure 3A). However, owing to the nature of the staining, it is also possible to visualize the breaking of the strands. In Figure 3B,C, hyphae were observed in the strands of the treated cigarette butts.

**Figure 4:**
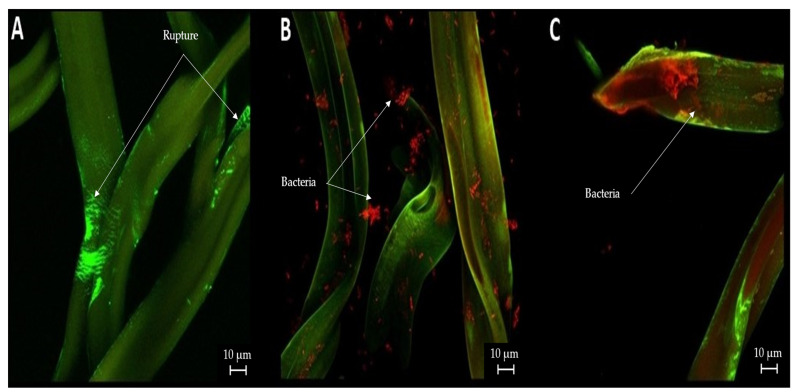
Confocal microscopy of cigarette butts: (**A**) smoked cigarette butt control (no biological treatment); (**B**) smoked cigarette butt treated with *B. cereus*; (**C**) smoked cigarette butt treated with *P. putida*.

The staining of the cigarette butt samples with propidium iodide and SYTO9 allowed the observation of bacteria in the treated samples. As shown in Figure 4A, corresponding to the controls, cellulose acetate threads were observed without the presence of microorganisms. On the other hand, the presence of microorganisms is shown in Figure 4B,C, which is highlighted in red. However, as in the previous case, owing to the nature of the staining, this area expands to the damaged areas of the samples (Figure 4A).

### 3.4. Scanning Electron Microscopy (SEM)

SEM visualization (Zeiss EVO MA10, Oberkochen, Germany) was performed to observe surface and morphological changes in the treated cellulose acetate cigarette butts with respect to the control.

SEM images revealed a high presence of fungal and bacterial material in the treated samples, as well as modifications in the morphology of the cigarette butts, especially those treated with fungi, where it was possible to observe small fractures and changes in the porosity of the material. It is also possible to visualize colonization through the presence of hyphae inside cellulose acetate fibers.

## 4. Discussion

### 4.1. Loss of Weight

The loss of weight observed by the degradation caused by the brown rot fungus *L. lepideus* may be due to the mechanism of degradation, which involves the use of hyphae first to start the biodegradation process, so this process tends to be incomplete. On the other hand, the fungus *P. ostreatus*, from which white rot is a corrosive type that uses enzymes such as ligninases, cellulases, and laccases, can break the structure of the molecule, allowing a greater degree of biodegradation [27,28].

The group of enzymes that causes the extracellular degradation of cellulose corresponds to cellulases, which are produced by hydrolytic systems of different microorganisms and are able to break β-glycosidic bonds. However, the biodegradation of cellulose acetate is inhibited by acetyl groups. Several studies have reported a weight loss of approximately 33% under standard composting conditions for fibers obtained from cotton and rice straw after 105 days under similar conditions [29]. Several studies have reported weight losses of approximately 35–44% in fibers obtained from cotton and rice straw under standard composting conditions after 105 days. The highest weight loss was observed after the thermophilic phase, which reached temperatures of approximately 65 °C. [29]. In this research, similar biodegradation percentages were achieved in just 31 days, demonstrating the efficacy of the decay rot fungi used. However, although a longer exposure time to both fungi could potentially increase the degradation percentage, this is not always guaranteed due to the presence of recalcitrant components, such as a crystalline structure of cellulose acetate, as well as chemical compounds, such as tar, found in CBs from different brands [30,31]. Moreover, it has been shown that the deacetylation process and the degradation rate are not linear; however, certain phases of the process, particularly at the beginning, may follow a linear trend, driven by the active growth of the fungi and their initial access to the substrate [29].

With respect to the bacterial action of CA, the synergistic action of the enzymes acitylesterase and endoglucanase has been reported in different studies, revealing the DS reduction capacity of a molecule of cellulose acetate. The accessibility of endoglucanases helps degrade the chains of cellulose acetate when its DS is low [32]. As reported by Haske et al., the acetylesterases are part of a group of enzymes that enables a reduction in cellulose acetate. However, these effects depend on the position of the acetyl group, as these enzymes do not achieve deacetylation of the DS over 1.8 [33]. The bacteria *B. cereus* and *P. putida* are Gram-positive and Gram-negative bacteria, respectively, and these bacteria have been used as biodegrading agents of different toxic and polluting compounds, such as herbicides, polyethylene, and hydrocarbons, among others [34]. When the loss in weight of cigarette butts treated with bacteria was analyzed, this capacity was confirmed. A reduction in the acetate compounds can be accomplished by cellulolytic enzymes present in these bacteria. The cellulases produced by these organisms are usually of three main types: endoglucanases (1,4-D-glucan-4-glycanohydrolases), exoglucanases (celodextrinases), and cellobiohydrolases (1,4-Dglucan-cellobiohydrolases) [2].

In the case of *B. cereus* bacteria, no significantly different results were found among the brands of treated cigarette butts. The cellulolytic enzymes produced by this genus are mainly endo-β-1,4-glucanase, exoglucanase, and β-glucosidase, which can degrade the acetate compound, breaking the bonds of different positions of the chain [35].

*P. putida* is one of the most commonly used bacteria for the degradation of plastics and aromatic compounds because of its several metabolic mechanisms, and several studies have shown that this biodegrading capacity is effective [36]. Therefore, it is not difficult to infer that this process can also promote the biodegradation of compounds derived from cellulose. However, the results of the analysis of weight loss revealed meaningful differences for each brand of cigarette butts used, which could indicate that there could be differences in their composition, which may affect the degree of degradation performed by this bacterium.

A study with the microorganisms *Rhizobium meliloti* and *Alcaligens xylosoxidans* reported weights of 34% and 23%, respectively, in the membranes of cellulose acetate after 150 days of incubation at 53 °C and 60% moisture content [37]. Bacteria and organisms derived from the soil during the degradation of the cigarette butts decreased in weight by 64% for the cellulose filters and 17% for the cellulose acetate filters after 157 days under average Mediterranean climatic conditions, corresponding to 17 °C and a cumulative rainfall of 124 mm. This finding proves that the percentage of weight loss depends to a large extent on the microorganisms used.

Accordingly, the idea that fungi, as well as bacteria, have an enzymatic battery that allows biodegradation of the cellulose acetate compound is reinforced, since although greater degradation of the compound in the treatments involving bacteria is observed, it is important to mention several studies that confirm the use of microbial consortiums that act synergistically to attain the intended goal as the best way to achieve degradation [38]. It is important to mention that biodegradation systems are complex; although degradation is based on enzymatic activity, to confirm the function of a specific enzyme, additional studies are needed, such as the deactivation of genes related to enzymatic activity and monitoring of metabolites produced by the degradation of the material to corroborate its role in decomposition.

### 4.2. Fourier-Transform Infrared Spectroscopy (FTIR)

FTIR analysis confirmed the degradation of CA acetylation in cigarette butts. In other studies, the characteristics of the peaks in the 1750–1000 cm^−1^ zone (-C=O/acetate; C-O/carboxylic acid; C-O-C/dialkyl) may be used as a qualitative method for this DS [11]. In addition, the peaks shown in the 3000 cm^−1^ zone (alcohols, aromatic compounds) may be related to nicotine and aromatic oils in cigarettes [39].

Owing to the ability of fungi to penetrate, it is possible to observe the effects of their ability to colonize cigarette butts. The deacetylation of cellulose acetate has been shown to be key when degrading this material. The decrease in the functional groups corresponding to the aromatic groups, as well as the alkanes, indicates another advantage for the use of rot fungi: the possibility of degrading damaging compounds such as nicotine and heavy metals in cigarettes.

Regarding what was observed in the FTIR spectra of the bacteria *B. cereus* and *P. putida*, in some cases in “Figure 2” of the “Results” section, increased spectra are shown compared with their respective controls; these increases may result from the production of compounds generated by both bacteria under certain conditions. *B. cereus* can produce acetyl methyl carbinol, together with polyhydroxyalkanoates and melanins, among others [40]. Similarly, *P. putida* can produce some of these compounds, and several studies have used this species as a yielder of PHA polymers known as bioplastics [41].

During the degradation of aromatic hydrocarbons, several microorganisms produce biosurfactants, which are compounds that promote interactions between microorganisms and insoluble substrates. These may be made up of polysaccharides, proteins, lipopolysaccharides, and lipoproteins, among others [42]. Therefore, when nicotine and aromatic oils are present in cigarette butts, the presence of such compounds cannot be disregarded in the analysis in future studies, and these compounds can be measured by the Wilhelm method.

### 4.3. Confocal Microscopy

The images obtained via confocal microscopy corresponding to “Figure 3 and Figure 4” in the Results Section show the presence of fungi and bacteria in the cellulose acetate. However, owing to the nature of the markers used for both cases, it is also observed that since the propidium iodide and SYPRO markers are better attached to the damaged zones of the acetate strand, a notorious presence of these may be observed in the controls (following a different pattern than the one observed in biological cells) for any damage caused by heat, either by the activity of smoking or not. The full degradation of CA could not be observed throughout the confocal microscopy study because this technique was principally focused on detecting fungal fibers and bacterial cells attached on the fibers instead material degradation. However, several studies have shown that during the first month, CA degradation is greater. Therefore, it is necessary to study this material for a longer period to visualize the real degradation capacity of this material [5], with different treatments performed.

The evolution shown by the cigarette butts during this research shows a remarkable weight loss and degradation of the CA compound due to the microbial degradation process, where microorganisms already attached to the cigarette butt surface start to secrete specific enzymes that biodegrade the CA into smaller molecular substances or fragments, thus removing part of the acetyl group and facilitating the effective degradation of the material [43]. WRFs and BRFs have been the subject of numerous studies confirming their ability to biodegrade various compounds [44,45]. These fungi possess unique extracellular ligninolytic and oxidative systems, including enzymes such as peroxidases, laccases, and carbohydrate-active enzymes (CAZymes), which enable them to transform or degrade various contaminants because of their ability to interact with multiple types of substrates [46,47]. Likewise, CA-degrading cellulase-producing bacteria, including strains of *Pseudomonas* (evaluated in this study) and *Neisseria sicca*, have contributed to the weight reduction and degradation of the compound, reflecting results obtained in previous studies and underscoring the potential of these bacteria to facilitate effective biodegradation. The *N. sicca* strains also produce acetyl esterase, suggesting a more efficient mechanism for the deacetylation of CA [29].

### 4.4. Scanning Electron Microscopy (SEM)

**Figure 5:**
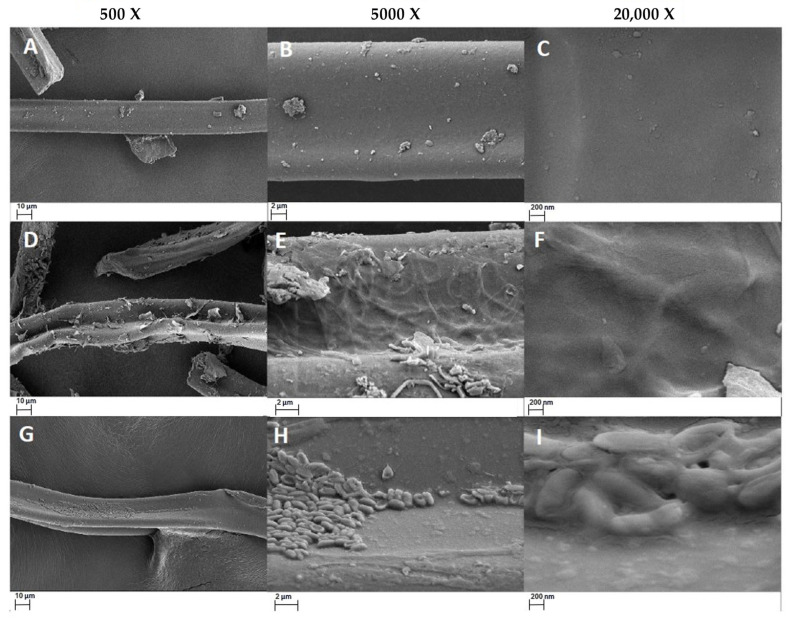
SEM micrographs of the cellulose acetate samples analyzed, corresponding to the control with no biological treatment (**A**–**C**), fungal treatment (**D**–**F**) and bacterial treatment (**G**–**I**), at four magnifications: 500×, 2000× (just for the control “**B**”), 5000× and 20,000×.

Scanning electron microscopy (SEM) was used in our study to provide detailed evidence of the morphological alterations induced by biological treatments with fungi and bacteria in cigarette butts, clearly contrasting with the controls (Figure 5).

The SEM images of the fungal-treated samples (Figure 5D–F) revealed a high presence of fungal material as well as minor cracks and changes in the porosity of the material. These surface changes suggest active degradation of cellulose acetate, facilitated by the penetration of hyphae within the fibers, confirming that colonization by *P. ostreatus* and *L. lepideus* allows degradation or biodeterioration by extracellular enzymes [48].

On the other hand, samples treated with bacteria (Figure 5G–I) presented remarkable bacterial clustering, forming robust biofilms on the surface of the cigarette butts. This biofilm is a prominent aspect of the biodegradation of synthetic materials by bacteria because it facilitates degradation by increasing the contact area between the bacteria and the surface of the material, thereby accelerating surface decomposition [49].

These results demonstrate that the changes on the surface of the cigarette butts are indicative of enzymatic activity and degradation of the material by *P. ostreatus*, *L. lepideus*, *B. cereus*, and *P. putida*, which, possibly through acetyl esterases, cellobiohydrolases, and cellobiases, could have contributed to the deacetylation of cellulose acetate and chain scission. Acetyl esterases remove acetyl groups and increase the accessibility of cellulose to cellulolytic enzymes such as endoglucanases, cellobiohydrolases, and cellobiases that catalyze the hydrolysis of the β-1,4-glycosidic bonds linking the glucosyl units of cellulose, ultimately transforming them into glucose, allowing the final products to be used as substrates by microorganisms for efficient biodegradation and colonization of the material [33,50]. The weight loss data and the observations of degradation and colonization of the cigarette butts obtained through SEM correlate, indicating that the extensive microbial colonization observed is associated with increased biodegradation of the material, as reflected in the reduction in its mass, highlighting the promise of biological approaches for cigarette waste management. Furthermore, these results suggest new possibilities, such as possible interactions between *P. ostreatus, L. lepideus, B. cereus*, and *P. putida*, which could increase the efficiency of this process. Although each microorganism individually is capable of breaking down cellulose acetate, the potential synergy between them could facilitate more complete and efficient degradation [51]. For example, a previous study demonstrated the effective degradation of poly(adipate-co-butylene terephthalate) (PBAT) using microbial co-culture systems [52]. Another study reported that the synergistic use of the brown-rot fungus *Fomitopsis pinicola* and the bacterium *Ralstonia pickettii* achieved degradation of 1,1,1-Trichloro-2,2-bis (4-chlorophenyl) ethane (DDT) [53]. Finally, a consortium of *Pseudomonas* and *Bacillus* strains grew synergistically in the presence of polyethylene terephthalate (PET) and bis(2-hydroxyethyl) terephthalic acid (BHET) as sole carbon sources through degradation of these compounds [54]. Thus, further research is needed to better understand how these interactions can be manipulated to improve the biodegradation processes of cigarette butts.

## 5. Conclusions

This study evaluated the biodegradative capacity of the fungi *P. ostreatus* and *L. lepideus* as well as the bacteria *B. cereus* and *P. putida* on cigarette butts incubated with these microorganisms for 31 days in an effort to develop effective methods for their treatment. After incubation, the results revealed that both fungi and bacteria demonstrated significant abilities to degrade the cellulose acetate present in cigarette butts. Bacterial treatments, particularly with *B. cereus*, were more effective, achieving up to 33% weight loss, while fungal treatments reached 30%. Analytical techniques such as FTIR and microscopy, both confocal microscopy and scanning electron microscopy (SEM), confirmed structural and morphological changes in the material, highlighting the effectiveness of biological treatments through microbial colonization and growth. Additionally, the findings suggest that neither the cigarette butt brand nor the presence of toxic compounds generated by smoke significantly influenced the biodegradation process, underscoring the robustness of the microorganisms used and indicating that the primary limiting factors for cigarette butt biodegradation may be more closely linked to environmental and microbial interactions rather than to brand-specific additives or toxic compounds. Differences in efficacy between the fungal and bacterial treatments studied suggest that each organism may rely on distinct enzymatic mechanisms, which could guide future research to optimize biotechnological strategies and help clarify which factors most effectively enhance cigarette butt degradation. Overall, this study provides potential candidates for waste management, reduces the environmental impact of cigarette butts, and underscores the need for further exploration and application of biological solutions to address the challenges posed by persistent waste.

## Data Availability

The original contributions presented in the study are included in the article; further inquiries can be directed to the corresponding author.
